# Unipolar versus bipolar hemiarthroplasty for displaced femoral neck fractures: a systematic review and meta-analysis of randomized controlled trials

**DOI:** 10.1186/s13018-015-0165-0

**Published:** 2015-01-24

**Authors:** Zhiwei Jia, Fan Ding, Yaohong Wu, Wei Li, Haifeng Li, Deli Wang, Qing He, Dike Ruan

**Affiliations:** Department of Orthopaedics, Navy General Hospital, Beijing, China; Department of Orthopaedics, Wuhan Pu’Ai Hospital, Tongji Medical College, Huazhong University of Science and Technology, Wuhan, China

**Keywords:** Femoral neck fractures, Arthroplasty, Hemiarthroplasty, Randomized controlled trials, Systematic review, Meta-analysis

## Abstract

**Background:**

Whether bipolar hemiarthroplasty (BH) for displaced femoral neck fractures has benefit over unipolar hemiarthroplasty (UH) remains controversial. We conducted a systematic review and meta-analysis of randomized controlled trials (RCTs) to evaluate the relative effects between BH and UH.

**Methods:**

A systematic literature search (up to April, 2014) was conducted to include RCTs comparing BH with UH for displaced femoral neck fractures. Two authors independently assessed methodological quality of the included studies and extracted data. Surgical information and postoperative outcomes were analyzed.

**Results:**

A total of 10 RCTs including 1,190 patients were indentified. Our results demonstrated that BH was associated with similar or better outcomes in hip function, hip pain, and quality of life while with a higher cost compared with UH. Moreover, there were no significant differences between BH and UH with regard to operation time, blood loss, blood transfusion, hospital stay, mortality, reoperation, dislocation, and complications. BH could significantly decrease the incidence of acetabular erosion at 1 year follow-up compared with UH (RR = 0.24, 95% confidence interval (CI) = 0.06 to 0.89, *P* = 0.03), but no significant difference was observed at 4 months, 2 years, and 4 years follow-ups.

**Conclusions:**

Based on the current evidence, BH is not superior to UH in terms of surgical information and postoperative results. Despite similar or better clinical outcomes compared with UH, BH with a higher cost could not decrease long-term acetabular erosion rate.

## Introduction

Femoral neck fracture is a common fracture in senior patients, which can decrease mobility and increase mortality [1]. There are many options for these fractures including internal fixation, hemiarthroplasty, and total hip arthroplasty [2]. Among these procedures, hemiarthroplasty has become the most preferred treatment option for surgeons according to the surveys [1-3]. There are two types of options, including unipolar hemiarthroplasty (UH) and bipolar hemiarthroplasty (BH), when using a hemiarthroplasty. In comparison to UH, BH has an additional inner bearing between the stem and the endoprosthetic head component. In theory, this design would decrease acetabular erosion, decrease protrusion, and decrease dislocation, as well as maintain joint stability and improve hip function [4,5]. However, whether UH or BH is preferable for the patient population remains uncertain [6-13].

Although several randomized controlled trials (RCTs) on this topic have been published, these studies showed inconclusive and controversial results [14-23]. The previous systematic reviews demonstrated that further well-designed RCTs were needed to draw a definitive conclusion, indicating the scientific evidence was still insufficient [24,25]. However, many RCTs have been published [14,18,19,21,23,26], since the latest meta-analysis conducted in 2010 [25]. The need remains for strong evidence including the recent RCTs to make a more precise estimation [27]. Therefore, the objective of this meta-analysis was to include all available RCTs and to evaluate the relative effects between UH and BH for displaced femoral neck fractures.

## Materials and methods

This study was performed and reported following the Preferred Reporting Items for Systematic Reviews and Meta-Analyses (PRISMA) statement [28]. This study was approved by the ethical review committee of Navy General Hospital and Wuhan Pu’Ai Hospital.

### Search strategy

A comprehensive search (up to April, 2014) without restriction on language was independently conducted by two reviews (ZJ and FD) through the databases of Pubmed, Embase, and Cochrane Library. The MeSH terms (hip fractures, femoral neck fractures, arthroplasty, hemiarthroplasty, hip prosthesis) and multiple keywords (unipolar, bipolar, arthroplasty, hemiarthroplasty, replacement, prosthesis, fractur*) were used to ensure inclusion of all possible studies. These terms were connected by the Boolean operators “AND” and “OR”. Additionally, the reference lists of included articles and relevant reviews were also examined for potential studies.

### Inclusion and exclusion criteria

The eligible articles should meet the following inclusion criteria: (1) RCTs comparing UH with BH; (2) patients with displaced femoral neck fractures; (3) at least one of the following main clinical outcomes: surgical information and postoperative outcomes. Studies were excluded if they had any of the following characteristics: (1) reviews, abstracts, letters, or meeting proceedings; (2) patients with immature skeleton, delayed union, nonunion, previous surgery, or pathological fractures; (3) duplicate reports of an earlier trial or no interest outcomes reported.

### Data extraction

Two reviewers (ZJ and FD) independently extracted the data from all eligible RCTs with the use of a standardized data recording form. Disagreements were resolved by discussion, a third review (YW) was consulted for the final decision when necessary. The data of interest included the following categories: (1) study characteristics such as year of publication, sample size, age, gender, and follow-up duration; (2) surgical information including operation time, blood loss, blood transfusion, and hospital stay; (3) primary outcomes comprising functional scores, pain, range of motion (ROM), 6-min walk, quality of life and cost (4) secondary outcomes including mortality, reoperation, dislocation, complications, and acetabular erosion. In addition, complications were sorted into four categories, including implant-related complications (periprosthetic fractures, prosthesis loosening, dislocation, etc.), cardiovascular and cerebrovascular complications (cardiac arrest, myocardial infarction, acute cardiac arrhythmia, cerebrovascular accidents, pulmonary embolism, deep venous thrombosis, etc.), local complications (wound infection, wound hematoma, incision rupture, heterotopic ossification, etc.), and general complications (pneumonia, urinary tract infection, bedsore, gastrointestinal bleed, acute renal failure, etc.).

### Risk of bias assessment

Two reviewers (ZJ and FD) independently assessed each of the included study. Disagreements were resolved by means of discussion, with arbitration by a third reviewer (HL), when differences of opinion remained. The risk of bias of the included studies was evaluated using the bias assessment tool recommended by the Cochrane Handbook for Systematic Reviews of Interventions (version 5.1.0) [29]. For each trial, the risk of bias was categorized as low risk, high risk, or unclear risk. Bias assessment was carried out using RevMan 5.2.10 software (Cochrane Collaboration, UK).

### Statistical analysis

For each included study, mean differences (MD) and confidence intervals (CIs) were calculated for continuous outcomes, while risk ratios (RRs) and 95% CI were calculated for dichotomous outcomes. Heterogeneity across trials was assessed with use of both the chi-square (*χ*^2^) test and the *I*-squared (*I*^2^) test. Statistical heterogeneity was considered significant when *P* < 0.10 for the *χ*^2^ test or *I*^2^ > 50% [30]. A random effects model was used to ensure that these studies represented a random sample of all potentially available studies [31]. Subgroup analysis was carried out according to specific complication categories and follow-ups. Sensitivity analysis was performed to test the strength and robustness of pooled results by sequential omission of individual studies. Publication bias was assessed using a funnel plot of the most frequently reported outcome. All reported *P* values were two-sided, and *P* < 0.05 was regarded as statistically significant. Statistical analyses were conducted by RevMan 5.2.10 software (Cochrane Collaboration, UK).

## Results

### Literature search

**Figure 1:**
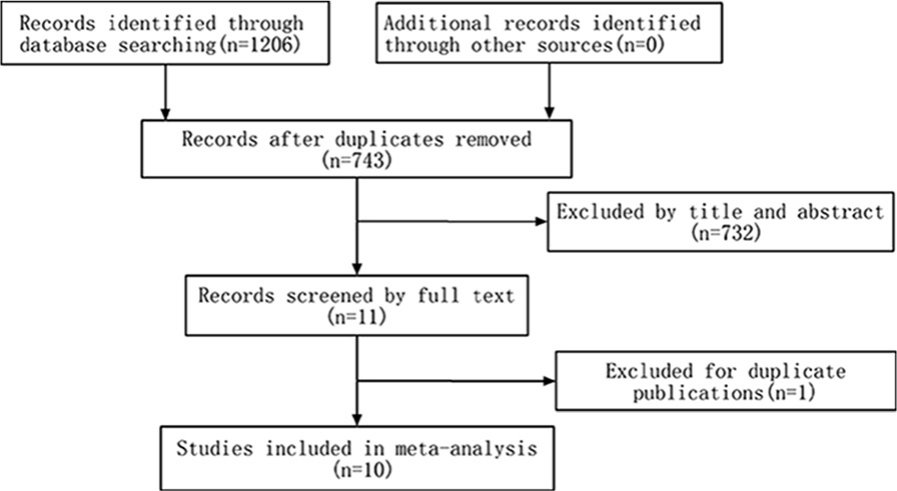
**The flow chart of study selection.**

The flow diagram of study selection is shown in Figure 1. A total of 10 RCTs comparing UH with BH for displaced femoral neck fractures were included in the present meta-analysis. These studies were from America, Australia, United Kingdom, Sweden, Egypt, Nepal, and India. All selected studies were published in English between 1995 and 2013. The sample size of the RCTs ranged from 40 to 261. A total of 1,190 patients involving 597 patients for BH and 593 patients for UH were identified. Table 1 summarizes the study characteristics of the included studies.

**Table 1:** **Study characteristics of the included studies**

**Author**	**Country**	**Study design**	**Patients**		**Mean age (y)**		**Gender (M/F)**		**Follow-up (months)**
			**BH**	**UH**	**BH**	**UH**	**BH**	**UH**	
Inngul [18]	Sweden	RCT	60	60	85.5	87.4	18/42	11/49	48
Stoffel [23]	Australia	RCT	133	128	82.9	81.9	89/172		12
Mishra [21]	Nepal	RCT	20	20	67		9/11	8/12	24
Abdelkhalek [14]	Egypt	RCT	25	25	63.5		16/34		24–72
Jeffcote [19]	Australia	RCT	24	27	80.1	81.4	6/18	6/21	24
Raia [22]	America	RCT	55	60	82.4	81.8	13/42	19/41	12
Davison [17]	United Kingdom	RCT	97	90	75	76	25/72	19/71	24–60
Cornell [16]	America	RCT	33	15	78	77.6	8/25	4/11	6
Calder [15]	United Kingdom	RCT	118	132	85	85	17/101	18/114	24
Malhotra [20]	India	RCT	32	36	65	68	18/14	20/16	9–47

*BH* bipolar hemiarthroplasty, *UH* unipolar hemiarthroplasty, *RCT* randomized controlled trial, *M* male, *F* female, *NA* not available.

### Risk of bias assessment

**Figure 2:**
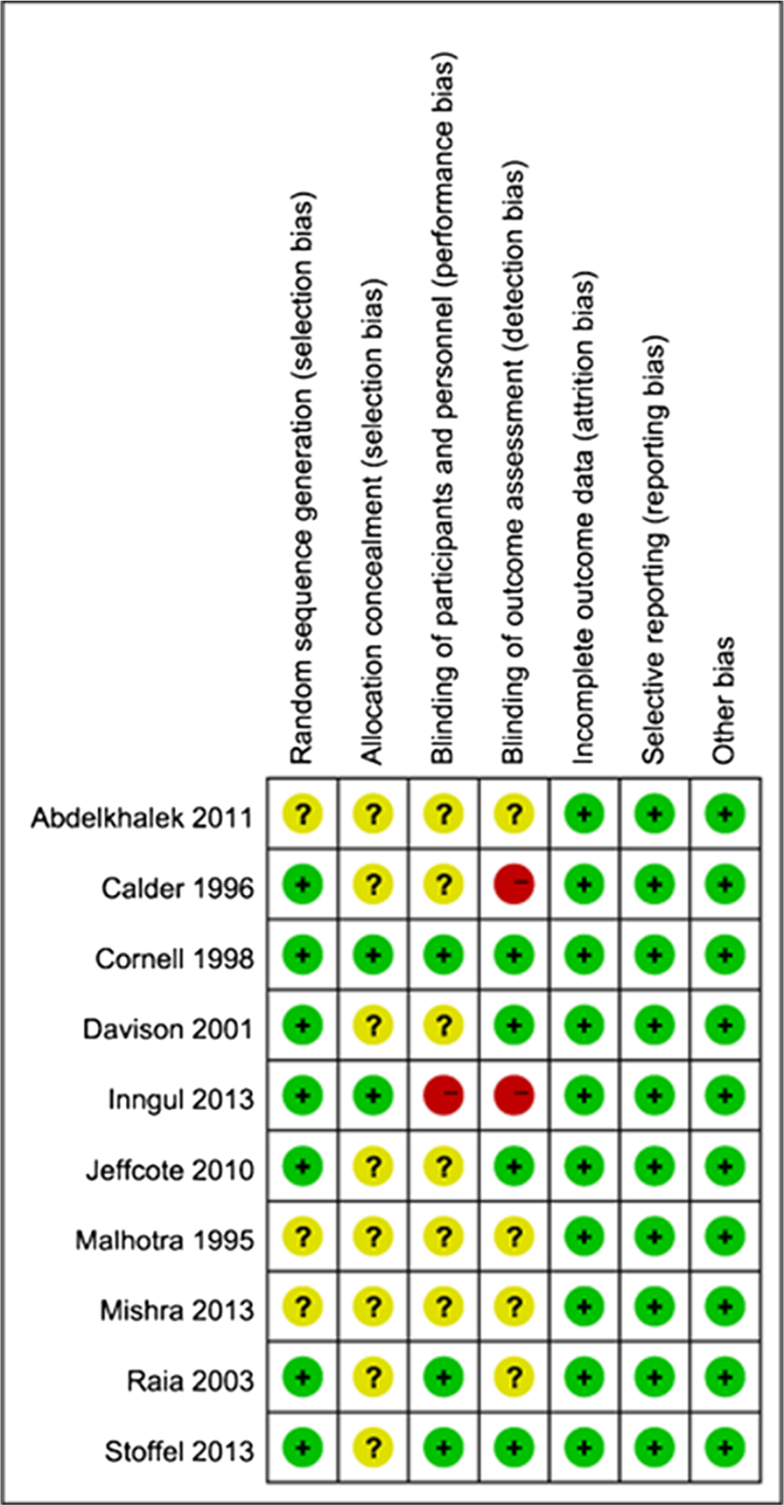
**Quality assessment of risk of bias in included studies.**

The risk of bias of the included studies is illustrated in Figure 2. Seven studies described adequate methods of random sequence generation [15-19,22,23], while the other three trials did not mention the methods of randomization [14,20,21]. Allocation concealment was well described in two trials [16,18] and was unclearly noted in the other eight studies [14,15,17,19-23]. Owing to the nature of the surgical trials, it was impossible to perform blinding of participants. However, patients were blinded to the type of prosthesis in three studies [16,22,23]. Additionally, four studies reported that outcome assessors were blinded [16,17,19,23]. Therefore the methodological quality of the included studies was low risk of bias.

### Surgical information

The summary of surgical information included operation time, blood loss, blood transfusion, and hospital stay. Due to standard deviation (SD) values of these outcomes were not shown, the meta-analysis could not be performed and a descriptive systematic review was conducted instead. Operation time was evaluated in three studies [16,18,20] and was shown no statistical difference between BH and UH. Regarding to blood loss revealed in four studies [16,18,20,22], there were no significances observed between the two groups in these studies. Only one study reported blood transfusion and showed that the proportion of blood transfusion was not obviously different [22]. Hospital stay was available in six studies and all these studies demonstrated no significant difference between UH and BH [15-17,20,22,23].

### Primary outcomes

#### Functional scores

The summary of functional outcomes is listed in Table 2. A total of nine studies assessed the hip function using functional scores [14-19,21-23]. Only one study revealed significant improvement in the BH group when compared with the UH group [14], while no significance was found in the other eight studies [15-19,21-23]. Various functional scores were used to assess hip function in the included studies, but only Harris hip score (HHS) was adopted in multiple studies [14,15,17-19,21,23]. Only two of them [21,23] reported the HSS as mean value and standard deviation, so they were included in the meta-analysis. The pooled results with no heterogeneity (*P* = 0.80, *I*^2^ = 0%) demonstrated that there was no statistical difference between the two groups (MD = −0.51, 95% CI = −4.43 to 3.42, *P* = 0.80).

**Table 2:** **Summary of postoperative clinical outcomes**

**Study**	**Hip functionality**			**Hip pain**	**Quality of life**
	**Functional scores**	**Six-minute walk**	**ROM**		
Inngul [18]	Similar	NA	Similar	Similar	Better
Stoffel [23]	Similar	Similar	Similar	Similar	NA
Mishra [21]	Similar	NA	Similar	Similar	NA
Abdelkhalek [14]	Better	NA	Better*	Better	NA
Jeffcote [19]	Similar	Similar	NA	NA	NA
Raia [22]	Similar	NA	NA	Similar	Similar
Davison [17]	Similar	NA	NA	NA	NA
Cornell [16]	Similar	Better	Better*	NA	NA
Calder [15]	Similar	NA	NA	Similar	NA
Malhotra [20]	NA	NA	Better*	Better*	NA

*Better* better outcome with statistical difference in bipolar hemiarthroplasty group, *Similar* similar result with no significance between groups, *ROM* range of motion, *NA* not available.

*P value is not available.

#### Hip pain

Seven studies reported hip pain [14,15,18,20-23]. Because the methods of pain assessment were different or standard deviation was not available, the pooled analysis could not be performed. Table 2 illustrates a summary of the pain outcomes. Five studies noted that there was no statistical difference between the two groups [15,18,21-23], whereas the other two studies found that BH was associated with better outcome than that in the UH group according to hip pain [14,20]. However, *P* value was not available in one of them [20].

#### Range of motion

ROM of the hip was assessed in six studies [14,16,18,20,21,23], while the results could not be pooled. The findings of the included studies are listed in Table 2. Among them, three studies indicated similar results between BH and UH [18,21,23]. The other three studies found better ROM in BH group while the *P* values were not provided [14,16,20].

#### Six-minute walk

Three studies evaluated 6-min walk test [16,19,23] (Table 2). Jeffcote et al. [19] revealed that BH was associated with a statistical improvement at 3 months but not at 12 and 24 months, and Cornell et al. [16] found statistical difference at 6 months. However, no significance was observed in the study by Stoffel et al. [23] during a 1-year follow-up.

#### Quality of life

Two studies reported the data of quality of life using SF-36 [22] and EQ-5D [18] (Table 2). Raia et al. [22] conducted a study with a 1-year follow-up and revealed that there was no statistical significance in quality of life according to SF-36 scores between groups. However, Inngul et al. [18] found that EQ-5D was generally higher in the BH group at the follow-ups with a significant difference at 48 months [18].

#### Cost

Two trials assessed the cost of prosthesis [16,23]. All these trials found that the bipolar unipolar implants are more expensive than the unipolar implants [16,23].

### Secondary outcomes

#### Mortality

All included studies reported postoperative mortality. However, it could not be extracted in three studies for the number of death was not available for each group [17,18,23]. The pooled analysis of the other seven studies including 622 patients [14-16,19-22] showed that there was no significant difference comparing BH with UH (RR = 0.92, 95% CI = 0.59 to 1.44, *P* = 0.71). No significant heterogeneity was found (*P* = 0.63, *I*^2^ = 0%).

#### Reoperation

Reoperation was evaluated in all the included studies [14-23]. There was no evidence of significant heterogeneity (*I*^2^ = 27%, *P* = 0.22) across the studies. The pooled results showed that no statistical difference was observed between groups (RR = 0.98, 95% CI = 0.42 to 2.27, *P* = 0.95).

#### Dislocation

All the included studies assessed prosthesis dislocation, whereas one of these studies provided the overall dislocation from both groups and did not report the number in each group [21]. Therefore, the other nine studies with 1,150 patients [14-20,22,23] was pooled to analysis and showed that no evidence of significant difference between the two groups (RR = 0.76, 95% CI = 0.30 to 1.93, *P* = 0.57; *I*^2^ = 0%, *P* = 0.99).

#### Complications

Complications were provided in all the included studies. Two of them did not report the exact number of complications in each group [21,22]. The pooled analysis was conducted in the other eight studies [14-20,23] and demonstrated that there was no statistical difference between the two groups in implant-related complications (RR = 0.84, 95% CI = 0.39 to 1.81, *P* = 0.66; *I*^2^ = 0%, *P* = 0.55), cardiovascular and cerebrovascular complications (RR = 1.33, 95% CI = 0.63 to 2.81, *P* = 0.45; *I*^2^ = 0%, *P* = 0.66), local complications (RR = 1.53, 95% CI = 0.71 to 3.33, *P* = 0.28; *I*^2^ = 0%, *P* = 0.68), and general complications (RR = 0.65, 95% CI = 0.28 to 1.49, *P* = 0.31; *I*^2^ = 0%, *P* = 0.34).

#### Acetabular erosion

**Figure 3:**
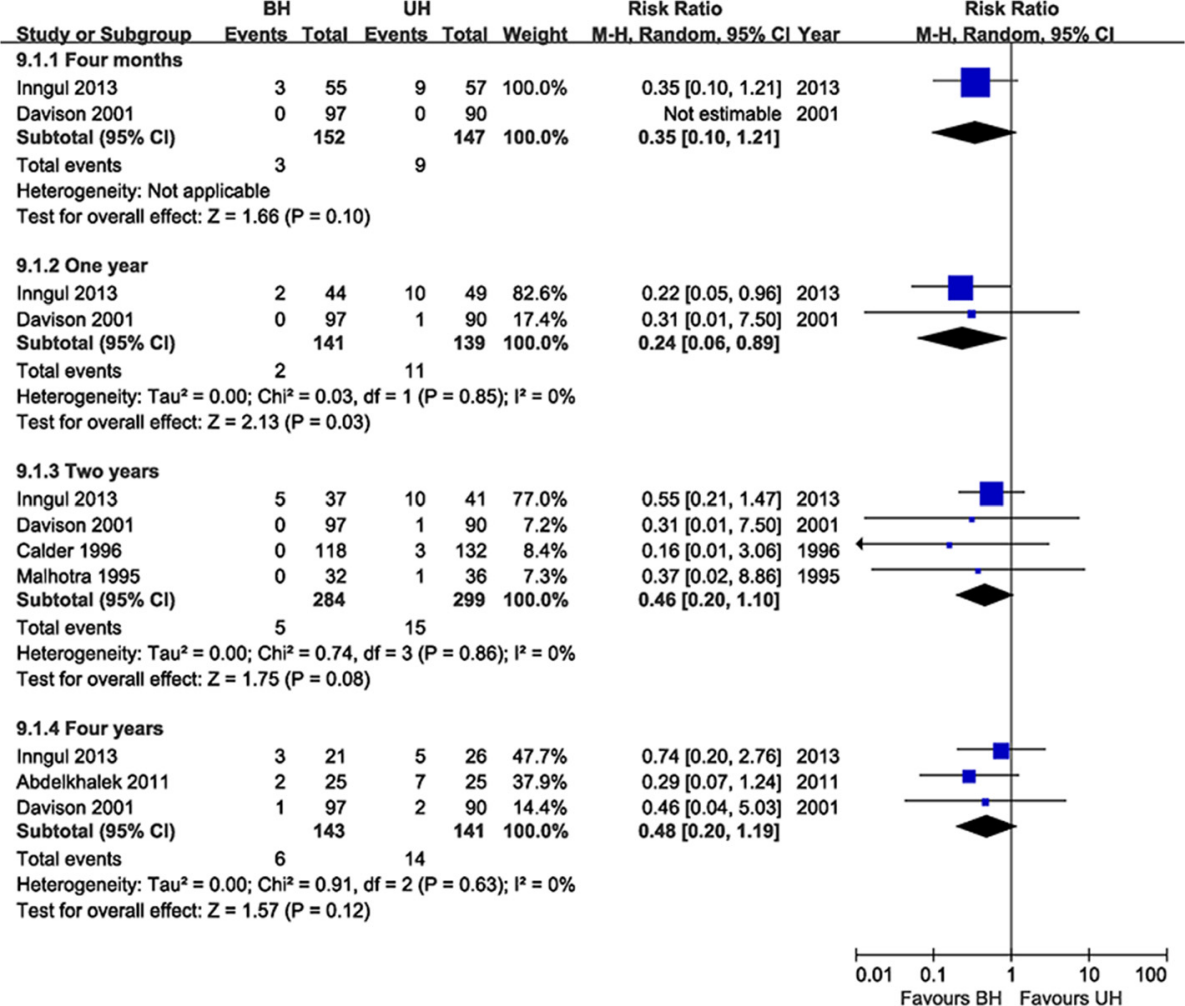
**Acetabular erosion.**

Six studies evaluated acetabular erosion [14,15,17,18,20,21], while the data was not available in one of these studies [21]. The other five studies were conducted for the pooled analysis [14,15,17,18,20]. Actetabular erosion in the BH group was significantly less than that in the UH group at 1 year follow-up (RR = 0.24, 95% CI = 0.06 to 0.89, *P* = 0.03), whereas no significant difference was detected between the two groups at postoperative 4 months (RR = 0.35, 95% CI = 0.10 to 1.21, *P* = 0.10), at 2 years (RR = 0.46, 95% CI = 0.20 to 1.10, *P* = 0.08), and at 4 years (RR = 0.48, 95% CI = 0.20 to 1.19, *P* = 0.12) (Figure 3).

### Publication bias and sensitivity analysis

A funnel plot of the studies reported reoperation was performed. It was illustrated that all studies were distributed evenly about the vertical, indicating minimal evidence of publication bias. Sensitivity analysis was conducted by omission of any individual study while no significant difference was observed in the pooled results.

## Discussion

Hemiarthroplasty, as an effective technique for displaced femoral neck fractures, could help early ambulation and satisfied function recovery and is increasingly performed by the surgeons [1-3]. However, controversy has persisted for a long time regarding the use of bipolar versus unipolar prosthesis. This study suggests that (1) BH is associated with similar or better improvement in hip functionality, hip pain, and quality of life compared with UH while with a higher cost and that (2) there are no significant differences between BH and UH with regard to operation time, blood loss, blood transfusion, hospital stay, mortality, reoperation, dislocation, and complications, and that (3) BH could not decrease acetabular erosion rate in the long term.

Compared with UH, bipolar prosthesis with an additional inner articulation has the theoretical advantages of less acetabular erosion and less dislocation [4,5]. This study demonstrates that the incidence of acetabular erosion in BH is less than that in the UH group at the follow-ups (Figure 3). These findings are consistent with the previous studies [6,13]. However, statistical difference was only noted at 1 year follow-up and the acetabular erosion rate increased at the later follow-ups with no significance observed (Figure 3). This may be because the bipolar articulation loses mobility with time and functions as a UH [18,26,32-35]. In addition, it should be recognized that this result should be interpreted with caution until confirmed by future studies, because the number of the pooled studies is small and the studies are of small sample size. Regarding to dislocation, it is not proved to be less comparing BH with UH in this study. Other studies have also failed to find this benefit [3,36,37]. However, the close reduction of bipolar head is more difficult than the unipolar prosthesis, and BH typically requires open reduction [38,39]. Moreover, BH could not increase the risk of operation in terms of surgical and postoperative results, including operation time, blood loss, blood transfusion, hospital stay, mortality, reoperation, dislocation, and complications. It may be demonstrated that BH is an alternative treatment as safe as UH.

It is also hypothesized that BH with lower acetabular erosion rate will produce a less painful arthroplasty and improve hip function and quality of life [4,5]. However, this meta-analysis failed to find the statistical difference in HSS score between BH and UH. Other hip functional scores, hip pain, and quality of life according to SF-36 and EQ-5D scores found inconsistent results (similar or better). It may be demonstrated that BH with a higher cost can achieve no less outcomes, so further studies are needed to perform the cost-effective analysis of BH versus UH. However, it should be recognized that these results were from the qualitative descriptive analysis of the available studies, not the meta-analysis, due to the heterogeneity among the studies. Therefore, more RCTs with the same outcome assessment scores are suggested and may help to get a more reliable conclusion.

The latest systematic review on this topic was published in 2010 and demonstrated that there was no significant difference in clinical outcomes between BU and UH [25]. However, there were several limitations in that study. It included both RCTs and quasi-RCTs, indicating a lower level of evidence of that study. Moreover, although seven studies were included in that study, only five studies were used for analysis, because two studies were conference abstracts without sufficient data. Therefore, there are several strengths in this meta-analysis. Firstly, more strict inclusion criteria were conducted. Only RCTs were included in this study, so the reliability of the results was ensured. Secondly, more RCTs published in recent years were included in this study, making the evidence much stronger. Thirdly, complications were further sorted. The potential bias risk from pooling all complications was decreased.

However, this meta-analysis also has several potential limitations. Firstly, publication bias which is common to all meta-analysis may be still unavoidable in this study. Secondly, various prostheses used in the included studies may induce related bias, whereas a subgroup analysis according to prosthesis type was not conducted due to insufficient data. Thirdly, different outcome measures were reported in the included studies, so a meta-analysis to statically strengthen the evidence could not be performed. Instead, a descriptive systematic review was conducted in these results.

## Conclusions

This systematic review and meta-analysis suggest that BH for displaced femoral neck fractures could not have benefit over UH in terms of surgical information and postoperative results, including operation time, blood loss, blood transfusion, hospital stay, mortality, reoperation, dislocation, and complications. BH may achieve similar or better outcomes compared with UH with respect to clinical outcomes, including hip functionality, hip pain, and quality of life. However, BH is associated with higher cost and could not decrease the incidence of acetabular erosion in the long term.
